# A miR-1207-5p Binding Site Polymorphism Abolishes Regulation of *HBEGF* and Is Associated with Disease Severity in CFHR5 Nephropathy

**DOI:** 10.1371/journal.pone.0031021

**Published:** 2012-02-02

**Authors:** Gregory Papagregoriou, Kamil Erguler, Harsh Dweep, Konstantinos Voskarides, Panayiota Koupepidou, Yiannis Athanasiou, Alkis Pierides, Norbert Gretz, Kyriacos N. Felekkis, Constantinos Deltas

**Affiliations:** 1 Molecular Medicine Research Center and Laboratory of Molecular and Medical Genetics, Department of Biological Sciences, University of Cyprus, Nicosia, Cyprus; 2 Medical Research Center, University of Heidelberg, Mannheim, Germany; 3 Department of Nephrology, Nicosia General Hospital, Nicosia, Cyprus; 4 Department of Nephrology, Hippocrateon Hospital, Nicosia, Cyprus; 5 Department of Life and Health Sciences, University of Nicosia, Nicosia, Cyprus; Universidade de Sao Paulo, Brazil

## Abstract

Heparin binding epidermal growth factor (HBEGF) is expressed in podocytes and was shown to play a role in glomerular physiology. MicroRNA binding sites on the 3′UTR of *HBEGF* were predicted using miRWalk algorithm and followed by DNA sequencing in 103 patients diagnosed with mild or severe glomerulopathy. A single nucleotide polymorphism, miRSNP C1936T (rs13385), was identified at the 3′UTR of *HBEGF* that corresponds to the second base of the hsa-miR-1207-5p seed region. When AB8/13 undifferentiated podocytes were transfected with miRNA mimics of hsa-miR-1207-5p, the HBEGF protein levels were reduced by about 50%. A DNA fragment containing the miRSNP allele-1936C was cloned into the pMIR-Report Luciferase vector and co-transfected with miRNA mimics of hsa-miR-1207-5p into AB8/13 podocytes. In agreement with western blot data, this resulted in reduced luciferase expression demonstrating the ability of hsa-miR-1207-5p to directly regulate HBEGF expression. On the contrary, in the presence of the miRSNP 1936T allele, this regulation was abolished. Collectively, these results demonstrate that variant 1936T of this miRSNP prevents hsa-miR-1207-5p from down-regulating HBEGF in podocytes. We hypothesized that this variant has a functional role as a genetic modifier. To this end, we showed that in a cohort of 78 patients diagnosed with CFHR5 nephropathy (also known as C3-glomerulopathy), inheritance of miRSNP 1936T allele was significantly increased in the group demonstrating progression to chronic renal failure on long follow-up. No similar association was detected in a cohort of patients with thin basement membrane nephropathy. This is the first report associating a miRSNP as genetic modifier to a monogenic renal disorder.

## Introduction

The inherited monogenic glomerulopathies is a genetically and phenotypically highly heterogeneous group of conditions. Even in specific monogenic diseases, the exact molecular pathomechanism underlying the variable expressivity is rarely well understood. This heterogeneity is exemplified by the observation that not all patients who develop chronic kidney disease (CKD) due to a primary genetic cause will proceed to end-stage kidney disease (ESKD). In such diseases, glomerular defects that include but are not limited to the glomerular basement membrane, the glomerular endothelium and the podocytes can alter the kidney's filtration barrier integrity and lead to an adverse outcome in patients. A subset of glomerular defects emerging from germinal mutations in specific genes or are acquired are directly reflected on podocytes, which may lose their structural integrity and functional properties [1], [2].

Microscopic hematuria (MH) of glomerular origin can be a benign condition persisting for life or can be the starting point of a progressive process that may lead many years later to proteinuria and decline of renal function resulting in CKD or ESKD [3]. A prime example is thin basement membrane nephropathy (TBMN), where patients in the same family who bear an identical heterozygous mutation in either the *COL4A3* or *COL4A4* gene that encodes for the α3 or α4 chain of collagen type IV respectively, may follow a quite diverse disease course. In recent studies on a large cohort of patients we showed that a small percentage of patients will remain for life with benign isolated MH; however a larger fraction of patients will proceed to proteinuria and CKD. Overall 15–20% of patients will have an even worse course and reach ESKD at ages after 50 years of age. In fact, nearly 50% of patients after 50 years will require hemodialysis or a renal transplant [4].

Similarly, in another recently revisited C3 glomerulopathy that is caused by mutations in the *CFHR5* gene which plays a role in the regulation of the alternative pathway of complement activation, nearly all patients present with MH since childhood while they may also develop macroscopic hematuria as a response to infections of the upper respiratory tract. A subset of patients will remain stable but about 15%, predominantly males will develop proteinuria and CKD or ESKD [5]. Female patients appear to have a milder disease progression and according to our recently published work, 14/18 patients who reached ESKD were males. This variable expressivity might be explained by a host of factors including genetic modifiers through yet unknown molecular mechanisms. MicroRNA (miRNA) regulation of gene expression could be one of these factors.

The role of miRNAs in processes such as maturation of the mammalian kidney was recently established by the podocyte-specific inactivation of Dicer, the RNAse III endonuclease responsible for miRNA maturation, in mice [6], [7], [8]. Podocyte foot processes were consequently depleted, while apoptosis commenced. The affected animals initially developed albuminuria followed by glomerular sclerosis and tubulo-interstitial fibrosis with acute renal disease progression and eventually death of mice by 6–8 weeks. The pathological phenotype was completed by proteinuria, glomerular basement membrane abnormalities and mesangial expansion, assimilating a congenital glomerulopathy. This proves that miRNAs have a fundamental role in regulating kidney physiological development; hence they must have a role in renal disease as well.

miRNAs belong to the most abundant class of small RNAs in animals. It is a recently discovered class of eukaryotic, endogenous, non-coding RNAs that play a key role in the regulation of gene expression. When mature they are short, single-stranded RNA molecules approximately 21–23 nucleotides in length, and they are partially complementary to one or more messenger RNA (mRNA) molecules [9]. Their main function is to down-regulate gene expression by inhibiting translation or by targeting the mRNA for degradation or deadenylation [10]. The mature miRNA mainly acts by targeting a miRNA recognition element (MRE) on an mRNA's 3′UTR and binding on it through a Watson-Crick base-pairing manner [11]. miRNA target recognition properties depend on its ‘seed region’, which includes nucleotides 2–8 from the 5′-end of each miRNA [12]. Base-pairing between the 3′-segment of the miRNA and the mRNA target is not always essential for repression, but strong base-pairing within this region can partially compensate for weaker seed matches or enhance repression [13].

In general there are two main mechanisms by which miRNAs can be involved in disease pathogenesis. A mutation on the miRNA itself can render it the primary causative gene. On the other hand, a miRNA can be indirectly involved in disease expression if the gene it targets is defined as the causative gene. The only evidence of a miRNA itself being the primary causative gene came from the work of Mencia et al., in which they identified a point mutation in the seed region of miR-96 which causes autosomal dominant non-syndromic hearing loss [14]. An engineered mouse model with a mutation in the seed region of miR-96, presented a phenotype similar to the human disease confirming the primary role of miR-96 [15]. In contrast, a miRNA can be considered as secondary cause to the disease when a genetic variation alters the binding of that miRNA to a causal gene. Evidence for such mechanism was shown for miR-24 when a point mutation that altered its binding to SLITRK1 gene was identified in patients with Tourette syndrome [16]. Similarly, point mutations on REEP1 which is a causative gene for hereditary spastic paraplegia were found on the binding sites of two miRNAs (miR-140 and miR-691) [17], [18].

Involvement of miRNAs in inherited diseases is not limited to those two mechanisms. Evidence suggests that miRNAs can act as disease modifiers as a result of genetic variations on the precursor molecules or the miRNA-target binding sites. Single nucleotide polymorphisms (SNPs) can affect all states of the miRNAs' synthesis (pri-, pre-, and mature) and alter the miRNA biogenesis or function. Variations that alter the biogenesis of miRNAs were associated with predispositions to various diseases including congenital heart disease [19], schizophrenia [20], papillary thyroid carcinoma [21] and others. Despite that, it should be noted that genetic variations within the pre-miRNAs and specifically within the seed-region are rare and comprise less than 1% of the miRNA-related SNPs [22].

MicroRNA associated single nucleotide polymorphisms (miRSNPs) found on miRNA target sites within 3′UTRs of mRNAs, are relatively common. A miRSNP can eliminate or weaken the binding of a miRNA to its target site or increase the binding by creating a perfect sequence match to the seed of a miRNA that normally is not associated with the given mRNA, provided that both miRNA and mRNA share the same tissue of expression. In both cases the result will be a significant alteration in protein levels. There are currently three databases available (Patrocles, dbSMR and PolymiRTS) that compile SNPs on the mRNA 3′UTR region of human and mouse genes that create or destroy miRNA binding sites [23], [24], [25].

Here we hypothesized that miRSNPs might act as genetic modifiers predisposing to a milder or more severe disease on the background of a primary inherited glomerulopathy, such as TBMN and/or CFHR5 nephropathy. The initial bioinformatics *in silico* analysis that was followed by extensive DNA re-sequencing revealed one such polymorphism, SNP C1936T in the 3′UTR of *HBEGF* (rs13385, 3′UTR+1006), that corresponds to the second position of the seed region of miRNA hsa-miR-1207-5p. Its significance was demonstrated by functional studies in undifferentiated cultured podocytes and by association studies in two cohorts of patients. Specifically, in the presence of a mimic for miRNA hsa-miR-1207-5p there was down regulation of the HBEGF expression, judged by western blot analysis. This was corroborated by the use of luciferase sensor constructs of both alleles, where the 1936T allele demonstrated abrogation of miRNA binding. Most interestingly, the 1936T allele was shown to act as a genetic modifier, as it was genetically associated with a higher risk for progression to severe renal disease in the presence of a primary glomerulopathy, C3 glomerulonephritis.

## Methods

### Patients

Patients who participated in this study were diagnosed with TBMN or CFHR5 nephropathy and were all shown to have inherited mutations in either the *COL4A3*/*COL4A4* genes or the *CFHR5* gene respectively, in heterozygosity. All participants were informed by the clinicians and signed a consent form. The project is approved by the Cyprus National Bioethics Committee. A total of 232 anonymous DNA samples from our DNA bank served as controls.

TBMN patients originate from 16 large Cypriot families. Seventy-eight of 103 patients are heterozygous for the G1334E–*COL4A3* mutation, 19 of 103 are heterozygous for the G871C–*COL4A3* mutation and 6 of 103 are heterozygous for the c.3854delG–*COL4A4* mutation [26]. Due to the slow disease progression patients with “mild disease” (see below) and younger than 48-yo (born before January 1963) were excluded. The CFHR5 nephropathy group was comprised of 45 male and 33 female patients (born before January 1975), all sharing a common exons 2–3 heterozygous duplication in *CFHR5* gene [5]. Pedigrees and analytical clinical data have been published in detail elsewhere [26], [27]. For both, TBMN and CFHR5 cohorts, “mildly” affected patients are those having only microscopic or macroscopic hematuria episodes (but no CKD) or hematuria plus low grade proteinuria (<400 mg/24 hrs, but no CKD). “Severely” affected patients are those having hematuria plus proteinuria >500 mg/24 hrs or hematuria plus proteinuria plus CKD or ESKD. CKD was defined as an elevated serum creatinine over 1.5 mg/dl. Patients with remittent or borderline proteinuria were excluded. Patients with a concomitant renal disease (*e.g.*, over five years diabetes, diabetic nephropathy, vesicoureteral reflux) or at the extreme of body weights (outside ±2 SD of the cohort mean) were also excluded.

### Gene selection

In accordance with our hypothesis we searched for SNPs in the 3′UTR region of genes and specifically around the putative target regions of respective miRNAs. Eighty five genes were selected based on a wide spectrum of criteria. Candidate genes belong to four general categories based on their glomerular expression, their involvement in monogenic glomerular diseases, whether they were previously associated with a polygenic disease that presents secondary glomerulopathy and other genes expressed in the kidney or elsewhere that were found to be important for renal function or are closely related to genes selected in other categories. Podocyte specific genes, such as NPHS1, NPHS2 or PDPN are considered as good candidates, while polygenic diseases include diabetes, systemic lupus erythematosus, IgA nephropathy, glomerulonephritis and hypertensive nephrosclerosis. Published data regarding kidney or glomerulus specific gene expression microarray experiments enriched the candidate gene list, thus including genes coding for transcription factors, activators, structural proteins etc. In addition, genes implicated in tubular disease like *PKD1* and *PKD2* were also included.

### miRNA target prediction analysis

Candidate gene names were imported into miRWalk algorithm (www.ma.uni-heidelberg.de/apps/zmf/mirwalk) and prediction of miRNA target sequences on their mRNA 3′UTR was performed using 7 nucleotides as the minimum seed number. A multiple comparison using 4 additional algorithms was performed for filtering purposes, each one working based on different sets of properties among mRNA-miRNA targeting; TargetScan, miRanda, miRDB and RNA22. In search for polymorphic variants by DNA sequencing around the miRNA target sequences, our attention was restricted only to pairs of miRNA-mRNA targets that were predicted by all five algorithms and gave a p-value<0.05. This p-value was automatically calculated by the miRWalk algorithm by using Poisson distribution and depicts the distribution of the probability of a miRNA 5′-end sequence to be randomly paired with a given 3′UTR mRNA sequence.

### DNA sequencing analysis of target regions

DNA sequencing of predicted target regions was performed using BigDye™ V3.1 chemistry on an ABI Prism™ Genetic Analyzer (Applied Biosystems, California USA). Sequencing primers (all supplied by MWG, Ebersberg, Germany) were designed to flank the target region but also included an additional 300 bp on average on each side. Sequence electropherograms were obtained from the ABI Sequencing Analysis™ V5.2 software (Applied Biosystems, California, USA) and sequences were imported into BioEdit™ Software to be aligned against a reference sequence with ClustalW algorithm [28]. SNPs that were identified in positions other than the predicted ones were evaluated using the miRanda tool (http://www.microrna.org) and cross-referenced with initial predictions.

### Expression reporter system constructs

To evaluate the binding efficiency of miRNAs onto predicted target sequences, the pMiR-REPORT™ miRNA Expression Reporter Vector System (AMBION, Texas, USA) was used. For the case where we identified a SNP in the 3′UTR region, each allele was obtained with a polymerase chain reaction (PCR) amplification from two patients, each one homozygous for either allele and primers were designed to introduce a *Spe*I and a *Hind*III restriction enzyme sites to be cloned into the pMiR-REPORT™ Luciferase vector. For rs13385, the insert included 297 bp of *HBEGF* 3′UTR that flanked the SNP. Ligation products were transformed into competent DH5a *E. coli* cells (Takara, Japan). Insert verification included a restriction reaction with *Spe*I and *Hind*III and sequencing using 100 ng of DNA.

### Transfection of AB8/13 podocytes

The AB8/13 undifferentiated podocyte cells, supplied by Dr Moin A. Saleem [29], were incubated at 33°C at 5% CO_2_ and cultured in RPMI medium, supplemented with 10% Fetal Bovine Serum (FBS) (Invitrogen, California, USA), 1% of 100 units/ml Penicillin/Streptomycin (Invitrogen, California, USA) and 1% Insulin-Transferrin-Selenium (Invitrogen, California, USA). For the luciferase reporter system experiments, AB8/13 cells were triply transfected with equal amounts of the pMIR-REPORT™ Luciferase and β-gal vectors and 25 nM of miScript™ hsa-miR-1207-5p mimic (QIAGEN, West Sussex, UK) or the AllStars™ Negative Control scrambled sequence LNA (QIAGEN, West Sussex, UK), using Lipofectamine 2000 (Invitrogen, California, USA). The β-gal vector was used for normalization. Every experiment was performed in triplicates in 6-well cell culture plates with the appropriate controls. Cells were harvested 12 h after transfection. The Dual-Light Assay™ Kit (Applied Biosystems, Caifornia USA) was used for the quantification of both luciferase and β-gal in an automated luminometer (Sirius, Berthold Detection Systems, Pforzheim, Germany). For western blot experiments, AB8/13 cells were transfected with 25 nM of miScript™ hsa-mir-1207-5p mimics and inhibitors, as well as with AllStars™ Negative Control scrambled sequence LNA (QIAGEN, West Sussex, UK) for 16 hours.

### Western blot experiments

AB8/13 cells were lysed in equal volumes of pre-heated 2xSDS loading buffer (Sodium Dodecyl Sulphate–125 mM Tris-HCl pH 6.8, 20% Glycerol, 2% SDS, 2% β-mercaptoethanol and bromophenol blue) and homogenized using a 2 ml syringe. Whole cell lysates were subsequently electrophoresed in a 12% SDS-Polyacrylamide gel. Gel transfer was held in a wet transfer system on Hybond Polyvinylidene Fluoride (PVDF–Millipore, Massachusetts, USA) membranes. Membranes were blocked with 5% non-fat dry milk in PBS/0.01% Tween20 for 1 hour at room temperature. Primary antibody was diluted in milk and added to the membrane for one hour. HBEGF protein was detected with the murine primary monoclonal antibody G-11 (SantaCruz Biotechnology, California, USA) at around 24 kDa. β-Tubulin was used as loading control by using the T-4026 primary antibody (SIGMA, Taufkirchen, Germany). As secondary antibody we used the rabbit anti-mouse antibody (SantaCruz Biotechnology, California, USA), conjugated with Horseradish Peroxidase (HRP). Proteins were detected using the Enhanced ChemiLuminescence (ECL) Plus Blotting Detection system (Amersham Biosciences, Buckinghamshire, UK) and were visualized by autoradiography on photographic film (KODAK X-OMAT, New York, USA). Band density was defined by ImageJ Software (http://imagej.nih.gov/ij).

### Real-Time PCR for miRNA detection

To examine the endogenously expressed levels of hsa-miR-1207-5p, total RNA enriched in small RNAs was isolated from AB8/13, HEK293 and SHSY-5Y neuroblastoma cells using the miRNeasy Mini Kit (QIAGEN, West Sussex, UK). MiRNA specific reverse transcription was performed with the miScript™ Reverse Transcription Kit (QIAGEN, West Sussex, UK). Real-Time PCR was performed on a Roche Lightcycler (Roche Diagnostics, Indianapolis, USA) using the miScript™ SYBR® Green PCR Kit (QIAGEN, West Sussex, UK), according to the manufacturers protocol. Detection of mature hsa-miR-1207-5p was accomplished using miScript™ Assay primers, supplied by QIAGEN. MiRNA enriched total RNA from human renal epithelial cells (HREpiC) was supplied by ScienCell (California, USA). Each experiment was performed twice in duplicates and miScript™ Hs_SNORA73A_1 small RNA was used as reference, with primers supplied from QIAGEN.

### Genotyping of miRSNP C1936T

Genotyping for the C1936T SNP was performed in all samples, either by direct re-sequencing (TBMN samples) or by restriction reaction analysis (CFHR5 and healthy control samples). For this purpose, a restriction recognition site for *Bsr*I was engineered in the forward PCR primer, by substituting the penultimate T by an **A** (Forward primer: 5′- CAA AGT GTA ACA GAT ATC AGT GTC TCC CCG TGT CCT CTC CC**A** G – 3′, Reverse Primer: 5′- GCT TTG CTA ATA CCT TCT CCA GAC TGT CCT CTG CTG CAC TGA -3′). The recognition site is created upon PCR amplification of the T allele. Subsequent sequencing analysis of selected samples confirmed the validity of this test. In addition, C1936T was also analyzed in the AB8/13 cell line by sequencing analysis, demonstrating a CC homozygous genotype.

### Statistical analysis

Genotyping results were statistically evaluated using two-sided Barnard's unconditional test of superiority, as it was shown that it is more powerful for 2×2 contingency tables with limited observations than conventional conditional tests [30], [31], [32]. The reported Wald statistic is the standardized difference between the two binomial proportions of each category [30]. For the analysis of contingency tables we used StatXact 9 (Cystat, Cambridge, MA, USA) [33], as suggested in a publication by Ludbrook J. (2008) [34]. In order to provide an open-source alternative for performing the Barnard's test, the “Barnard” package has been developed for the statistical scripting language R. The Barnard package is included in the Comprehensive R Archive Network (http://cran.r-project.org/web/packages/Barnard), where it is freely available for download and immediate use by anyone. C1936T was tested for Hardy-Weinberg equilibrium using Pearson's chi-square test in controls. Luciferase expression levels were analyzed using one-way non-parametric ANOVA after being normalized against β-gal expression levels. One-way ANOVA was also used to test densitometry results from western blot analyses, followed by Tukey post-testing.

## Results

### Bioinformatic analysis for identification of miRNAs as modifiers of glomerulopathies

Heritable monogenic glumerulopathies that present with MH display interfamilial and intrafamilial phenotypic heterogeneity, thereby suggesting the involvement of modifier genes in disease progression [35]. We herewith hypothesized the putative role of miRNAs as disease modifiers and we searched for functional polymorphic variants in the predicted target sites of miRNAs for genes expressed or located in the glomerulus. To this end, we guided our search for SNPs within the miRNA target sites of genes selected as described in Methods. Expression in podocytes, localization in the slit diaphragm and the glomerulus basement membrane rendered genes as good candidates for our study.

With the use of miRWalk (http://www.ma.uni-heidelberg.de/apps/zmf/mirwalk) and four other prediction algorithms (miRBase, TargetScan, miRDB and RNA22), we looked for validated miRNAs that target the candidate genes. We narrowed down the candidates of interest by selecting only miRNA-mRNA pairs that were predicted by all five algorithms (Table 1).

**Table 1:** Prediction results using five different miRNA-target prediction tools.

GENE	miRNA	RNA22	miRANDA	miRDB	miRWalk	TargetScan	SEED LENGTH	START	SEQUENCE	END	p-VALUE
PDPN	hsa-mir-485-5p	✓	✓	✓	✓	✓	8	1453	AGAGGCUG	1446	0.031
HBEGF	hsa-mir-212	✓	✓	✓	✓	✓	9	1584	UAACAGUCU	1576	0.0056
HBEGF	hsa-mir-132	✓	✓	✓	✓	✓	10	1584	UAACAGUCUA	1575	0.0014
HBEGF	hsa-mir-379	✓	✓	✓	✓	✓	8	1833	UGGUAGAC	1826	0.0223
FN1	hsa-mir-96	✓	✓	✓	✓	✓	8	8340	UUUGGCAC	8333	0.0169
FN1	hsa-mir-144	✓	✓	✓	✓	✓	9	8331	UACAGUAUA	8323	0.0042
GJA1	hsa-mir-495	✓	✓	✓	✓	✓	8	2244	AAACAAAC	2237	0.0261
PKD2	hsa-mir-183	✓	✓	✓	✓	✓	8	4768	UAUGGCAC	4761	0.0315
PKD2	hsa-mir-372	✓	✓	✓	✓	✓	9	4022	AAAGUGCUG	4014	0.008
PPARA	hsa-mir-223	✓	✓	✓	✓	✓	9	5877	UGUCAGUUU	5869	0.0314
SP1	hsa-mir-24	✓	✓	✓	✓	✓	7	5240	UGGCUCA	5240	0.2725
SP1	hsa-mir-31	✓	✓	✓	✓	✓	9	6960	AGGCAAGAU	6952	0.0197
SP1	hsa-mir-105	✓	✓	✓	✓	✓	7	5548	UCAAAUG	5542	0.2725
SP1	hsa-mir-155	✓	✓	✓	✓	✓	8	2560	UUAAUGCU	2553	0.0764
TJP1	hsa-mir-144	✓	✓	✓	✓	✓	8	6469	UACAGUAU	6462	0.0218

Ticks under algorithm names indicate the successful prediction of each miRNA-mRNA pair per prediction tool. “Start” and “end” columns state the exact position of the putative miRNA target region on the 3′UTR of the respective mRNA. Numbering refers to position from the start of the mRNA 3′UTR. For sequencing analysis, pairs that had p-values of less than 0.05 were selected.

### Identification of candidate SNPs by sequence analysis

**Figure 1:**
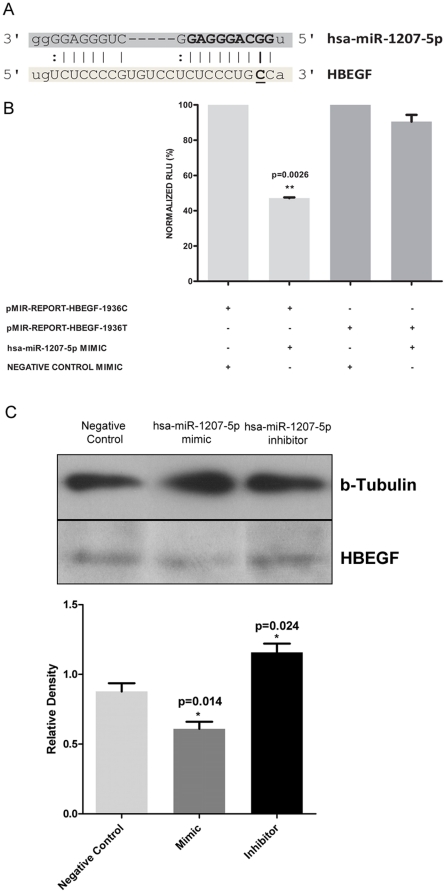
Effect of C1936T miRSNP in *HBEGF* gene on regulation by hsa-miR-1207-5p, in a podocyte culture system. A) Schematic depicting the position of miRSNP C1936T on the target region of hsa-miR-1207-5p on *HBEGF*. This miRSNP corresponds to the second base of the seed region of hsa-miR-1207-5p, underlined C. B) Normalized luciferase relative light units (RLUs) in AB8/13 cell lysates after transfection with sensor constructs. Co-transfection of the pMIR-REPORT-HBEGF-1936C with hsa-miR-1207-5p miRNA LNA mimics resulted in significant reduction of luciferase expression, with a p-value of 0.0026 using one-way non-parametric ANOVA test. In contrast, the pMIR-REPORT construct bearing the 1936T allele (pMIR-REPORT-HBEGF-1936T) abolished the hsa-miR-1207-5p binding site as demonstrated from the loss of RLU reduction. Results represent mean values of triplicates ± SEM. C) Western blot of HBEGF from AB8/13 cells after transient transfection with hsa-miR-1207-5p miRNA LNA mimics, inhibitors and the AllStars™ Negative Control scrambled sequence LNA. This is a representative of six experiments. Lower panel presents the statistical analysis of western blot densitometry results, normalized against the Negative Control. Values represent the mean ± SEM. Results illustrate the reduction of HBEGF protein levels at the presence of hsa-miR-1207-5p mimics (p = 0.014), while miRNA inhibitors significantly increased HBEGF levels (p = 0.024).

A segment of about 500–600 nts encompassing the miRNA binding site in the 3′UTR of selected genes, was re-sequenced in 103 patients with TBMN, classified as severe or mild. Table 2 summarizes the results of the sequencing analysis depicting the gene sequenced, the miRNA predicted to bind to the 3′UTR of that gene and the SNPs identified. Although various SNPs were identified in the group of patients sequenced, none was located on the predicted miRNA binding sites. However, a SNP was identified in the binding site of another miRNA that was originally excluded due to a lower significance compared to top candidates. Specifically, while sequencing around the hsa-miR-379 target site in the *HBEGF* 3′UTR, we identified a biallelic variation of C or T at position 1936 (C1936T) in the target region of hsa-miR-1207-5p which is also predicted to target *HBEGF*. The C1936T SNP is found at what corresponds to position 2 of the ‘seed’ region of hsa-miR-1207-5p (Fig. 1A) suggesting a possible elimination or severe compromise of the ability of this miRNA to bind on *HBEGF* mRNA.

**Table 2:** Results after re-sequencing of 103 samples with mutations in *COL4A3* or *COL4A4* genes and thin basement membrane nephropathy.

GENE	miRNAs	miRNA POSITION	PCR AND SEQUENCING PRIMERS	PREDICTION HITS	SNPs FOUND	NOTES
PDPN	hsa-mir-485-5p	1453-1446	5′- GTTAGGGCAGGTGGGATG -3′ 5′- TGTATGCGGCTGGTAAGTAG -3′	5/5	T1226AG1545AG1262A1251DEL-G	SNPs not on miRNA target sites
HBEGF	hsa-mir-132hsa-mir-212	1584-15751584-1576	5′- TGAACTGGAAGAAAGCAACA -3′ 5′- ACCCCTACATCCTGACCATAC -3′	5/5	None	No SNPs found
HBEGF	hsa-mir-379	1833-1826	5′- ACTCCTCATCCCCACAATCT -3′ 5′- CCCACCTCCAACCTTCTC -3′	5/5	C1936T	SNP found at neighboring position, which is target for hsa-miR-1207-5p
FN1	hsa-mir-96hsa-mir-144	8340-83328331-8322	5′- TTGGGATCAATAGGAAAGCA -3′ 5′- GAAGAGATGAAGTGACAAAAACC -3′	5/5	None	No SNPs found
PKD2	hsa-mir-183	4768-4760	5′- TCCAGGTTGAAAGTGAAAC -3′ 5′- CAGGGAAAGATAATAGGGAAGA -3′	5/5	None	No SNPs found
PKD2	hsa-mir-372	4022-4014	5′- TTCCCATGTGGCTCTACTCA -3′ 5′- AGACCCTCTCGTAAAGAAAACA -3′	5/5	G4003AG4210A	SNPs not on miRNA target sites
PPARA	hsa-mir-223	5877-5868	5′- GTTAGGGCAGGTGGGATG -3′ 5′- TGTATGCGGCTGGTAAGTAG -3′	5/5	None	No SNPs found
SP1	hsa-mir-31	6960-6951	5′- GACTTCCCCAAACCCAGA -3′ 5′- CACCCATCCCTTCCAGAG -3′	5/5	None	No SNPs found
TJP1	hsa-mir-144	6469-6462	5′- GGAGGGTGAAGTGAAGACAA -3′ 5′- GCATAGCCAGAAAGAACAGAA -3′	5/5	A6485C	SNPs not on miRNA target sites

Sequencing primers were designed to flank the predicted target sites and also include about 300 bp on either side. Prediction hits represent the number of tools that successfully predicted the miRNA-mRNA binding.

### Verification of functional significance by *in vitro* experimentation

In order to verify whether *HBEGF* is a true target of hsa-miR-1207-5p and that the presence of C1936T SNP alters the binding and regulation incurred by the miRNA, we performed luciferase ‘sensor’ assays. A segment of the *HBEGF* 3′UTR containing the 1936C (pMIR-REPORT-HBEGF-1936C) or 1936T (pMIR-REPORT-HBEGF-1936T) variant was cloned into the 3′UTR of the luciferase gene in pMIR-REPORT plasmid. Reporter plasmids and β-gal reference plasmid were co-transfected in AB8/13 podocyte cell line with either hsa-miR-1207-5p mimic or negative control mimics for 12 hours followed by luciferase and β-gal measurement. Co-transfection of hsa-miR-1207-5p mimics with the pMIR-REPORT-HBEGF-1936**C** resulted in significant reduction in luciferase expression (47.14%+/−0.42 SEM of normalized RLU relative to control) demonstrating that this miRNA directly binds on *HBEGF* 3′UTR region (Fig. 1B). In agreement, transfection of hsa-miR-1207-5p mimics in AB8/13 cells significantly reduced the endogenous levels of HBEGF protein as demonstrated by Western blot analysis at about 20% of total expression, while hsa-miR-1207-5p inhibitors boosted HBEGF levels by reducing the endogenously expressed miRNA levels (Fig. 1C). Densitometry of western blots revealed a significant decrease or increase of HBEGF levels on mimic or inhibitor transfection respectively. (Fig. 1C, lower panel).

On the contrary, in the presence of pMIR-REPORT-HBEGF-1936**T**, transfection of hsa-miR-1207-5p mimics did not significantly alter luciferase expression (90.56%+/−3.8 SEM of normalized RLU relative to control) in AB8/13 cells (Fig. 1B). Combined these results demonstrate that hsa-miR-1207-5p can directly regulate *HBEGF* expression and this regulation is abolished if there is a T nucleotide at position 1936 of *HBEFG's* 3′UTR.

**Figure 2:**
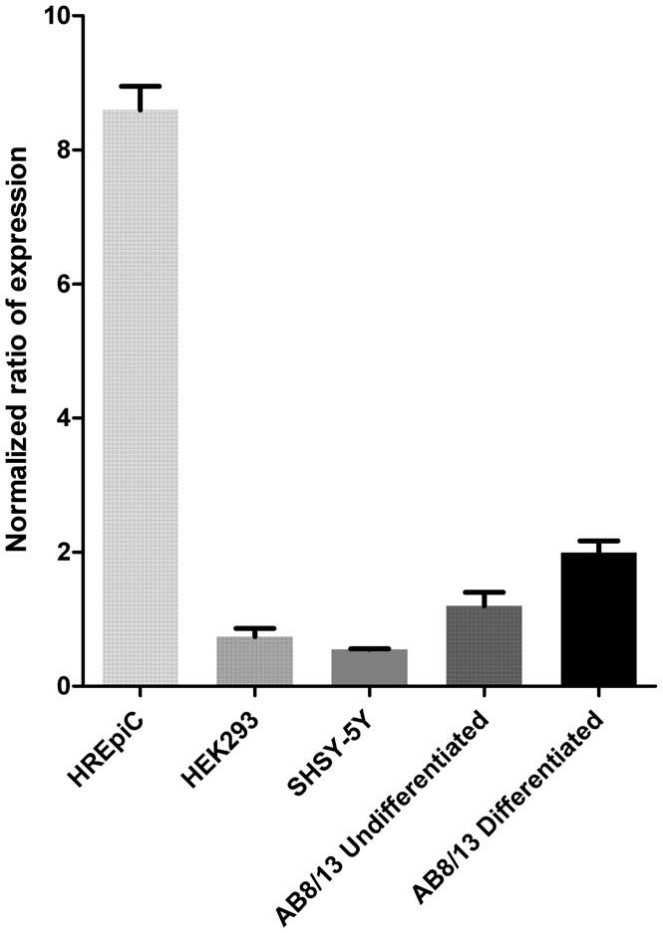
Expression analysis of miR-1207-5p in various cell lines. Relative expression analysis of mature hsa-miR-1207-5p levels in various cell types, as tested by miRNA specific Real-Time PCR experiments. Both AB8/13 differentiated and undifferentiated podocytes revealed significantly high expression levels of miR-1207-5p, compared to HEK293 and SHSY-5Y neuroblastoma cells. Further examination of miR-1207-5p levels in human renal epithelial cells (HREpiC), recorded 4-fold higher mature miRNA than the AB8/13 differentiated podocyte cell line. Results represent the mean of quadruplicate values ± SEM.

The hsa-miR-1207-5p is highly enriched in podocytes as demonstrated by miRNA specific Real-Time PCR experiments. Specifically, miR-1207-5p is expressed 2-fold higher in differentiated AB8/13 podocytes, compared to undifferentiated cells (Fig. 2). In addition, human renal epithelial cells express 4-fold higher miR-1207-5p than differentiated AB8/13 cells. Other cell lines, such as HEK293 and SHSY-5Y demonstrate limited expression levels of miR-1207-5p when compared to podocytes.

### Genotyping results

**Figure 3:**
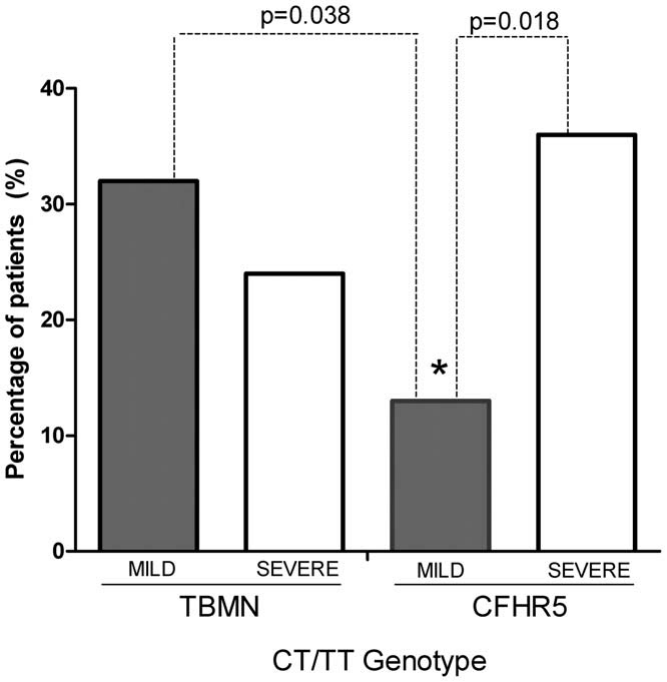
Mildly affected CFHR5 patients have lower occurrence of the 1936T allele. Graphical representation of both TBMN and CFHR5 nephropathy cohorts used in this study in relation to the number of CT and TT patients. Nephropathy patients with mild CFHR5 have significantly lower percentage of the CT genotype when compared with severe CFHR5 patients (p = 0.018), indicating a protective effect of the CC genotype. Statistical comparison between mild TBMN and mild CFHR5 patients demonstrated a significant underrepresentation of the 1936T allele in mild CFHR5 patients (p = 0.038). Mild TBMN patients did not differ from severe TBMN patients (p = 0.368). All statistical analyses were performed using two-sided Barnard's test.

We then tested the hypothesis that this variant may act as a genetic modifier in two cohorts of patients with inherited monogenic glomerulopathies. From 232 control subjects that were genotyped, 70% were CC homozygotes, 27% CT heterozygotes and 3% TT homozygotes. The control population obeys the Hardy-Weinberg equilibrium (p = 0.812), as tested by the Pearson's chi-square test. Sequencing analysis showed that 68.2% of patients having mild TBMN are homozygous for the C allele, 6.8% are homozygous for the T allele and 25%CT heterozygotes (Table 3). As regards patients with severe TBMN disease, 76.3% were CC homozygotes, 1.7% TT homozygotes and 22% CT heterozygotes. There was no statistical significance between the two groups, upon two-sided Barnard's testing (p-value = 0.368). We then tested a separate cohort of 78 patients diagnosed with CFHR5 nephropathy. Among 45 CHFR5 patients with milder disease progression, 86.6% are homozygous for the C allele, while the remaining are CT heterozygotes. In contrast, 63.6% of the 33 severely affected CFHR5 patients are CC homozygotes and 36.4% are CT heterozygotes. Barnard's test with a 95% confidence interval revealed an association between mild CFHR5 and the CC genotype, with a p-value of 0.018 and Wald statistic of −2.385 (Fig. 3).

**Table 3:** Genotype and allele frequencies in all study groups.

GENOTYPE/ALLELE	MILD TBMN(COL4A3/COL4A4 MUTATIONS)		SEVERE TBMN(COL4A3/COL4A4 MUTATIONS)		MILD CFHR5NEPHROPATHY		SEVERE CFHR5NEPHROPATHY	
CC	30	68.2%	45	76.3%	39	86.6%	21	63.6%
CT	11	25%	13	22%	6	13.4%	12	36.4%
TT	3	6.8%	1	1.7%	0	0%	0	0%
CT/TT	14	31.8%	14	23.7%	6	13.4%	12	36.4%

Under each group label, left columns demonstrate the number of subjects for each genotype and allele, while right columns the respective percentages.

Further grouping of TT homozygotes and CT heterozygotes, indicated a significant difference between mild CFHR5 patients and mild TBMN patients with a p = 0.0.038 after Barnard's test with a Wald statistic of 2.089. The corresponding frequencies did not differ significantly between severe CFHR5 and severe TBMN patients. Collectively, evidence suggests that the CT/TT genotype has no significant effect on the severity of TBMN, but it increases the risk for a severe outcome in patients with CFHR5 nephropathy, by 3.7 times.

**Figure 4:**
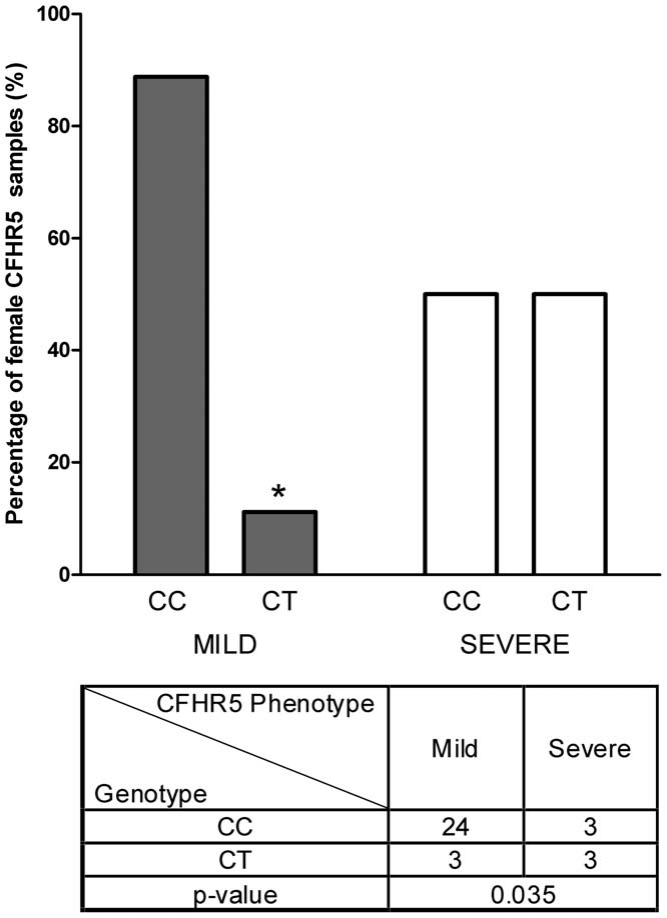
The 1936C/T *HBEGF* genotype is overrepresented in women affected with severe CFHR5. Comparison of C1936T genotypes in women manifesting CFHR5 nephropathy. Women are known to have a milder course of the disease when compared to men and this is also demonstrated when they are statistically compared with severe CFHR5 women as regards the C1936T SNP. The 1936C allele has a significantly lower representation in severe CFHR5 women, compared to mild women, thus suggesting a protective effect for this allele (p = 0.035).

A separate evaluation of women in our CFHR5 cohort, revealed significance between mildly and severely affected women with a p-value of 0.035 and a Wald statistic of −2.234 (Fig. 4). As women are known to have a milder course of the disease, it is 8 times more likely to have a severe phenotype if the patient is a female and a carrier of a CT/TT genotype.

## Discussion

The phenotypic heterogeneity and variable expression, exemplified as a broad spectrum of symptoms in a cohort of patients, is the norm in many monogenic disorders including renal conditions, such as glomerulopathies. The role of genetic modifiers, at least partly, is frequently invoked as they are hypothesized to act in symphony with a validated mutation in a single gene. For example, recent publications have reported several occasions where SNPs in genes confer a higher risk for progression of pathology, for example a SNP in the DKK3 gene or in the eNOS gene in polycystic kidney disease [36], [37], [38].

Having in mind recent advances in our understanding of molecular pathogenetic mechanisms, it is reasonable to expect that one other class of modifiers, among others, could be sequence variations on the target sites of miRNAs, in genes whose function relates to the disease under study. Inheritance of such variants is not expected to cause a disease in a Mendelian fashion; however their stochastic co-segregation with a primary disease-causing mutation may affect the risk for slower or faster progression of the phenotype. A mutation in a miRNA gene itself that is responsible for a Mendelian phenotype has been reported once, to our knowledge, whereas several publications report on the presence of pathology-associated variants in the target sites of known miRNAs. At the same time miRNAs can act as disease modifiers as a result of genetic variations on the precursor molecules. Specifically, SNPs may occur at the level of the pri-miRNA, pre-miRNA or mature miRNA. Such SNPs may affect either the biogenesis or the action of the mature miRNA, contributing to deregulation of target gene expression and consequently to disease development [39]. Notwithstanding this situation, most common are the miRNA-associated single nucleotide polymorphisms (miRSNPs) that are located in the miRNA target sites within the 3′UTRs of corresponding mRNAs. Several studies have identified associations of miRSNPs with complex trait diseases such as diabetes [40], asthma [41], Parkinson [42], hypertension [43], breast cancer with early age at onset [44] and others.

In this report, we show data according to which a reduction of hsa-mir-1207-5p binding ability on its target site in the 3′UTR of *HBEGF* due to the presence of C1936T SNP is associated with the severity of CFHR5 nephropathy in patients inheriting the pathogenic *CFHR5* gene duplication of exons 2–3. To our knowledge this is the first time a miRSNP is shown to be correlated with the phenotypic manifestation of a monogenic glomerular disease. Specifically, we showed that in a cohort of patients inheriting this CFHR5 nephropathy, the 1936C allele at the binding site for miRNA hsa-miR-1207-5p is associated with a less severe phenotype, as this is exemplified in patients who are protected from the development of high grade proteinuria and CKD (p = 0.018, Fig. 3). When concentrating on the subgroup of women, only three of 27 with mild disease inherited the T allele, compared to three of six women with severe disease (Fig. 4). This finding obtains particular significance in view of the fact that women follow a much milder course of disease compared to men, according to a previous work of our group [5], [27]. The exact mechanism by which *HBEGF* can alter disease phenotype is currently unknown and under investigation. However, we hypothesize that the role of *HBEGF* in proliferation and fibrosis of mesangial cells is very critical to this end. In support of its functional significance, we showed in cell culture experiments that the presence of the T allele eliminates the binding of the miRNA, thus resulting in higher HBEGF protein levels (Fig. 1).

We undertook a rather difficult approach to identify such a SNP, by collecting genes from the literature that are known to be involved in glomerular structure and function. In order to narrow down our search we utilized prediction algorithms in an attempt to extract the best candidate genes for sequence. In our case, the hsa-miR-1207-5p was predicted by two out of five algorithms to target *HBEGF*. Although not a top candidate, luckily enough hsa-miR-1207-5p target site is positioned nearby a site for an alleged good candidate miRNA (hsa-miR-379). As a result, we managed to prove the functional interaction of hsa-miR-1207-5p with *HBEGF*, because the cell culture experiments as well as the statistical evaluation in our cohort of patients supported its implication in gene regulation at post-transcription level. This case is a prime example where despite the improvement in bioinformatics tools and methods for predicting miRNA targets, some valuable information can still escape. The systematic approach we used however, along with the flexibility of our tools, enabled us to identify a functional SNP that otherwise would have been missed.

HBEGF belongs to the epidermal growth factor superfamily. It is also known as the Diphtheria toxin receptor since it is required for the surface binding of diphtheria toxin and entry into the cell [45]. This growth factor is expressed at high levels in podocytes, tubular epithelial cells and mesangial cells [46]. Several studies have emphasized the role of HBEGF in kidney function under normal or pathologic conditions. Ischemia/reperfusion (IR) injury was shown to be mediated by HBEGF, as reduction in expression of this protein had a protective effect in various IR models [45], [47]. As a member of a growth factor family, HBEGF can promote cellular proliferation in both mesangial and renal epithelial cells. Specifically, studies using renal proximal tubular cells revealed that proliferation in this cell type is mediated by HBEGF through an autocrine/paracrine mechanism which antagonizes the action of Src kinases [48]. In addition, *HBEGF* is expressed in mesangial cells and is involved in mesangial cells proliferation in glomerulonephritis and contributes to lesion formation in focal glomerular sclerosis through stimulation of mitogens at those sites [49], [50]. Similarly, HBEGF participates in renal fibrosis by regulating both TGF-β-mediated fibronectin expression and collagen expression in mesangial cells [51].

In humans, the miR-1207-5p is transcribed from the PVT1 locus on chromosome 8q24 [52]. The PVT1 gene is encoding for a non-translated RNA and has been found to be implicated in diabetic nephropathy and breast and colon cancer, in translocations related to Burkitt's lymphoma and associated with Hodgin's lymphoma [53], [54], [55], [56]. Interestingly, end-stage renal disease occurring in patients with diabetes type 2 has been associated with PVT1, while variants in the same gene were associated with ESKD in patients with type 1 diabetes [57], [58]. A recent study by Alvarez and DiStefano investigated PVT1 properties in depth and confirmed its high expression in mesangial cells, as well as proposed an up-regulation in the levels of the miRNAs emerging from PVT1 by elevated glucose in the mesangium [53]. These findings can supplement the results of our study and together are suggestive of novel roles for PVT1 influenced miR-1207-5p expression and translational regulation of HBEGF in terms of maintaining the physiological function of the mesangium or the glomerulus in general.

In conclusion, we presented evidence for the novel genetic modifier role of miRNA hsa-miR-1207-5p in predisposing patients with a monogenic recently described CFHR5 nephropathy, to more severe phenotype. The fact that all our patients shared the same exact germinal mutation probably was a factor that facilitated the identification of this modifier, as there was no confounding allelic heterogeneity. At the same time our data implicate *HBEGF* as the gene through which this miRNA exerts its effect. Further work at the cellular level and perhaps with the use of animal models will help elucidate in more detail the exact mechanism by which this hsa-miR-1207-5p/*HBEGF* pair plays its role. Notwithstanding our positive results and conclusions, it does not escape our attention that our cohorts are somewhat small. At this point in time it is impossible to enlarge the relevant cohort or to derive a new one before the passage of many years. Also, to our knowledge no patients have been diagnosed yet, of a different ethnic origin, as the C3 glomerulonephritis caused by a CFHR5 mutation appears endemic to Cyprus.
